# A Scintillator Array Table with Spectroscopic Features

**DOI:** 10.3390/s22134754

**Published:** 2022-06-23

**Authors:** Fabio Longhitano, Gaetano Elio Poma, Luigi Cosentino, Paolo Finocchiaro

**Affiliations:** 1 INFN Sezione di Catania, Via Santa Sofia 64, 95123 Catania, Italy; fabio.longhitano@ct.infn.it; 2 INFN Laboratori Nazionali del Sud, Via Santa Sofia 62, 95123 Catania, Italy; elio.poma@lns.infn.it (G.E.P.); cosentino@lns.infn.it (L.C.)

**Keywords:** radioactive waste sorting, radiation detection, hot-spot localization, scintillator array, SiPM array

## Abstract

In the framework of radioactive material handling, such as in radwaste sorting and segregation operations, the availability of a simple tool to quickly detect and locate gamma radiation spots can be quite convenient. Additional spectroscopic features, even with moderate energy resolutions, could provide a useful benefit. As a proof of principle for such a tool, we developed a gamma detector prototype featuring an array of 10 × 10 CsI(Tl) scintillators (1 × 1 × 1 cm^3^) providing readouts by means of a corresponding array of 6 × 6 mm^2^ silicon photo multipliers (SiPM). Such a detector table could be easily incorporated into a work desk for quick scanning of possibly radioactive objects. The proposed detector has a good counting efficiency and energy resolution, while the simulations and tests show interesting hot-spot localization capabilities.

## 1. Introduction

Nowadays, radioactive materials have to be handled in several fields and applications, such as in radwaste sorting and segregation [1,2], nuclear medicine and diagnostics [3,4], nuclear inspections and material accounting [5,6] and nuclear physics research. Well-defined prescriptions are given in each field concerning the material handling, radiation protection, safety and security. Many tools are commercially available to detect and monitor radioactivity, in particular gamma rays, which are the most ubiquitous form of radioactivity. More recently, compact gamma detection systems based on scintillators and silicon photo multipliers have been developed [7,8,9,10,11]. Most of these detection systems fall into one of the following two categories: environmental detectors and hand-held detectors. The former category refers to fixed systems, typically installed on racks, aimed at monitoring rooms or halls. The latter category includes detection systems that are actively used by an operator to check radiation levels or to scan objects or people for possible contamination.

What is proposed here is a gamma radiation detection system to be possibly incorporated as part of a work desk in order to perform continuous monitoring, and which can additionally locate hot-spots and perform a spectroscopic analysis on the materials under examination. For practical reasons, the prototype that is going to be described is a compact array of detectors with a 10 × 10 cm^2^ active area. Due to its modularity, it can be replicated in many different geometrical arrangements according to the specific application needs and requirements.

In the following, the characterization of the individual elements of the array will be discussed, as well as their collective behavior, showing that a larger system can be arranged in a (more expensive) configuration by tiling several arrays, but also with more sparsely distributed scintillators (cheaper) or even in a high spatial resolution line-scanner version.

## 2. Materials and Methods

The detector prototype that was developed, which is capable of detecting gamma rays with a pretty high efficiency, is an array of 10 × 10 CsI(Tl) scintillators with an individual size of 1 × 1 × 1 cm^3^. Each crystal is fully encased in white reflecting resin, apart from the bare face to be coupled with the photodetector. The chosen size is a tradeoff between the detection efficiency, position resolution, cost and compatibility with the photodetectors. Indeed, in order to keep the system compact and its cost and complexity low, we chose to employ MicroFC-60035-SMT-TA Silicon Photo Multipliers (SiPM) with a 6 × 6 mm^2^ active area, which is the maximum currently available on the market [12].

**Figure 1:**
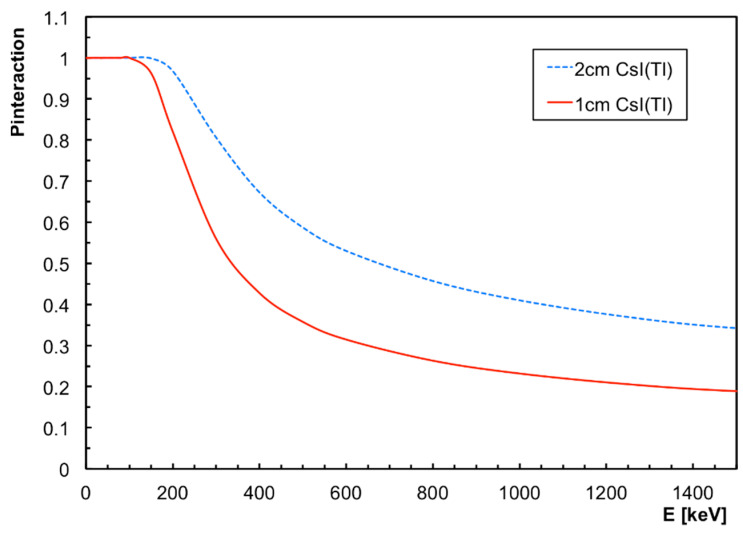
The probability of the interaction of a gamma ray in 1- and 2-cm-thick CsI(Tl) as a function of its energy.

The CsI(Tl) scintillator has been very well known for many years, and represents a comfortable solution for medium-resolution gamma counting and spectroscopy. It has a very good light yield of about 60,000 photons/MeV (for gamma radiation) [13], with a spectral emission centered around the wavelength of 550 nm, and is only slightly hygroscopic. Moreover, its convenient high density of 4.51 g/cm^3^ guarantees a good intrinsic detection efficiency, as shown in Figure 1 for 1- and 2-cm-thick CsI(Tl). The chosen thickness of 1 cm provides reasonable interaction probabilities of about 30% at 600 keV gamma rays and 20% at 1500 keV, which are not much worse than the 2 cm results, while being cheaper and more compact.

**Figure 2:**
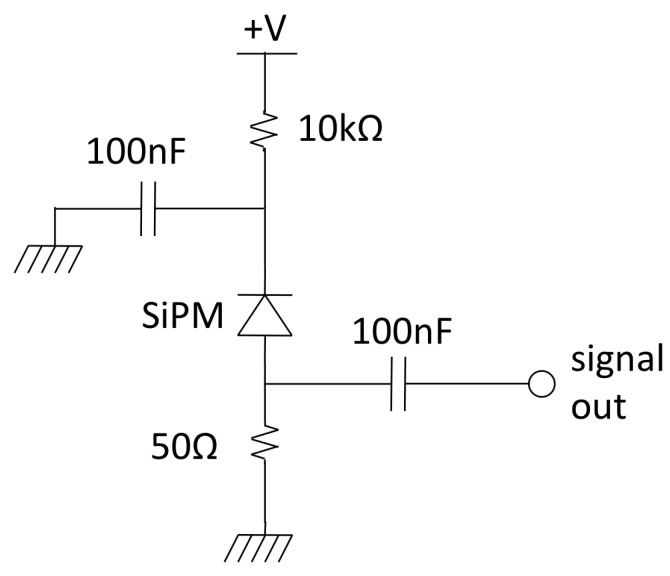
The polarization network and readout circuit of each SiPM, replicated on the electronic board for the 100 elements of the array.

**Figure 3:**
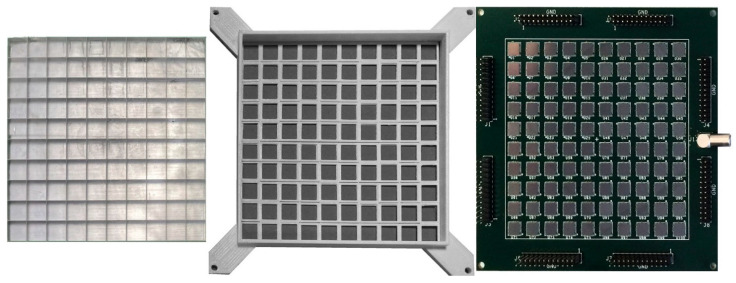
The detector components, from left to right: the scintillator array, the coupling frame mask and the SiPM array. On the four sides of the SiPM array board, the eight 26-pin dual header plugs for the output signals are visible, along with the bias voltage LEMO connector shown on the righthand side.

**Figure 4:**
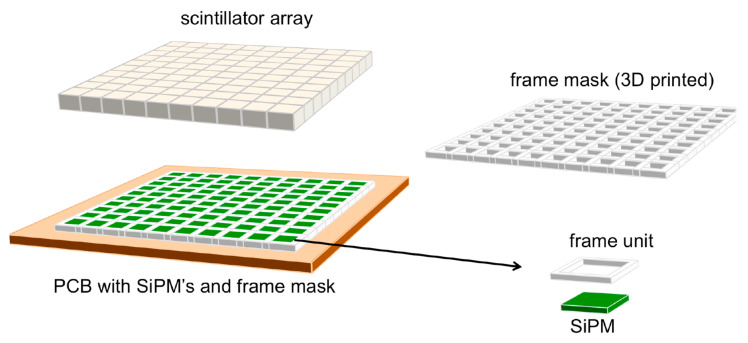
Sketch of the assembly scheme for the detector array. The white reflecting frame mask performs the mechanical alignment between the array of 6 × 6 mm^2^ SiPMs and the corresponding 10 × 10 mm^2^ scintillators.

The scintillation light is read out by means of a 10 × 10 array of SiPMs, assembled on a home-developed electronic board featuring all of the individual polarization networks and readout circuits, a scheme of which is shown in Figure 2. The SiPM bias voltage, which is unique for all the photosensors, is provided via a LEMO connector located on one side of the board. The bias for the normal operation was set at 27 V, which is about 2 V over the breakdown, as a tradeoff between the SiPM gain, in order to match the dynamic range of the digitizer, and its noise. In these operational conditions, the photon detection efficiency (PDE) for the CsI(Tl) light is about 20%. In total, 100 output signals are provided on eight 26-pin dual header plugs located on the four sides of the board, with each plug pair carrying a signal and ground. The array of scintillators was optically coupled to the SiPMs using Viscasil silicone optical grease, and the mechanical alignment was achieved by means of a suitable 3D-printed frame mask. The detector components, i.e., the scintillator array, the coupling frame mask and the SiPM array, are shown in Figure 3. Their assembling scheme is sketched in Figure 4. No front-end electronics were used, and the SiPM output signals were directly connected to a DT5740 digitizer produced by CAEN, which operates in charge integration mode [14]. Unfortunately, such a module can only handle up to 32 inputs; therefore, the tests described in this work were performed with a square sub-array of 5 × 5 elements. However, all other detector channels were individually tested and showed the same behavior.

The table can be operated in three modes: (i) as an overall gamma radiation counter, where the global counting rate is the sum of all individual counting rates; (ii) as a rough proximity imaging system with 1 cm^2^ pixels, which according to the application could be allocated sparsely instead of tightly packed like in our prototype, or could be masked with suitable collimators; (iii) as an array of spectroscopic detectors whose spectra can be summed on request to improve their statistics. The three operational modes are meant to be used progressively, first involving a coarse and quick counting measurement, then if a predefined threshold is overcome, this is followed by a slower hot-spot search. Finally, if hot-spots are found, a finer spectroscopic investigation is performed to look for specific isotopes or selective imaging is performed based on full-energy gamma detection with a reduction in the Compton background. Some applications could require the use of collimators in front of the detectors to achieve better imaging quality at the price of lower statistics. In order to explore this feature, measurements were performed with and without a collimator, with a small lead brick measuring 45 × 24 × 28 mm^3^ with a 3-mm-diameter hole.

## 3. Results

**Figure 5:**
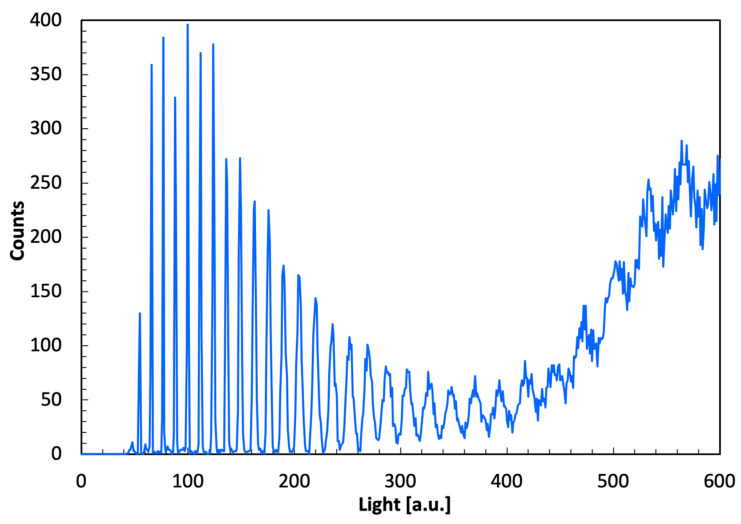
Lower-energy part of the spectrum obtained by exposing the detector to a ^22^Na source. Thirty very well resolved peaks corresponding to discrete numbers of scintillation photons detected by the SiPM are clearly observable.

In order to assess the single-photon detection capability of the SiPMs, the voltage supply was raised, operating at a higher gain to inspect the lower-energy part of the spectrum. In Figure 5, such a lower-energy part of the spectrum is shown for one element of the array, collected by exposing the detector to a ^22^Na source that produces 511 and 1275 keV gamma rays. Thirty very well resolved peaks, corresponding to discrete numbers of detected scintillation photons, are clearly observable in the plot.

Then, the spectroscopic response of the detector was tested using four different point-like laboratory gamma sources, namely ^241^Am, ^22^Na, ^137^Cs and ^60^Co, whose energy peaks and activity values are listed in Table 1.

**Figure 6:**
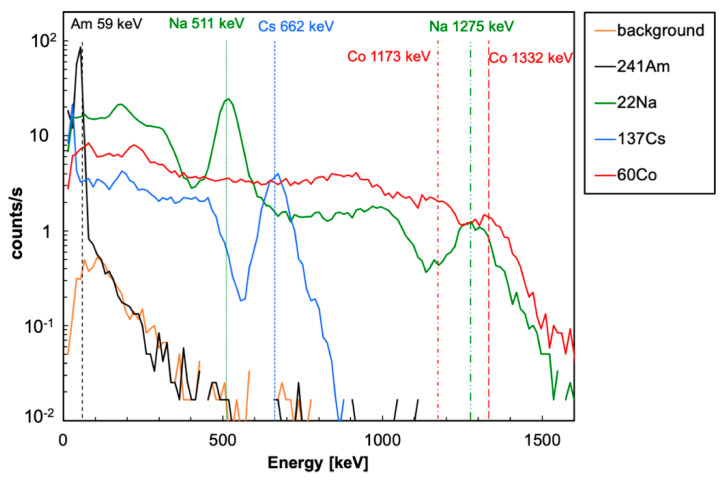
The energy spectra for the gamma sources of Table 1, placed at a 2 cm distance from the detector, and the background. Each spectrum was acquired in 120 s.

**Figure 7:**
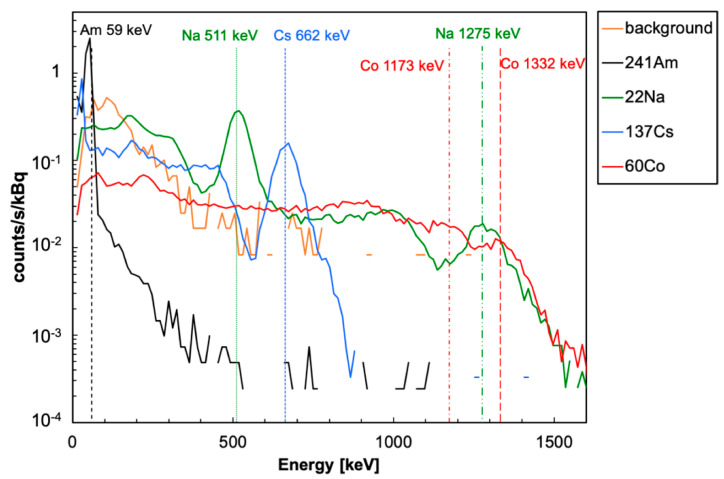
The energy spectra from Figure 6 normalized to the respective source activity.

Each source in turn was placed at a distance of 2 cm from the central detector, with a geometrical efficiency of about 2%, and the corresponding spectrum was acquired in 120 s. A spectrum without a source was also acquired to assess the contribution of the background. In Figure 6, the five resulting spectra are plotted for a linear calibration involving all six peak energies from Table 1. One can immediately see that the background contribution, with the chosen threshold set around 30 keV, is a few orders of magnitude below the real spectra. In order to make a more quantitative and reliable comparison, the plot shown in Figure 7 was produced, whereby the gamma spectra were normalized to each source activity. One can observe that in the adopted configuration (i.e., 2 cm source—detector distance), 1 kBq activity is still clearly visible above the background for all sources. It has to be remarked that being the two ^60^Co peaks close to each other, their resolution appears to be worsened by the partial superposition of their tails.

**Figure 8:**
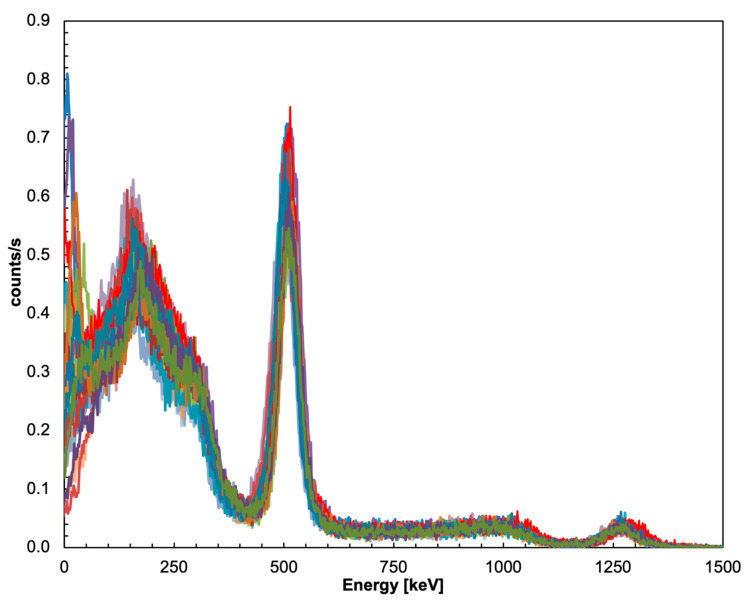
The 25 calibrated spectra from the configuration without the collimator.

**Figure 9:**
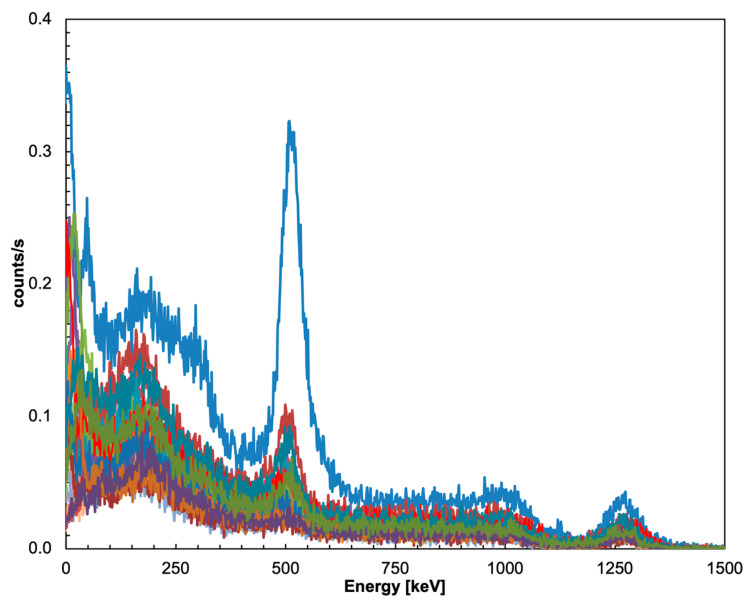
The 25 calibrated spectra in the configuration with the lead collimator.

**Figure 10:**
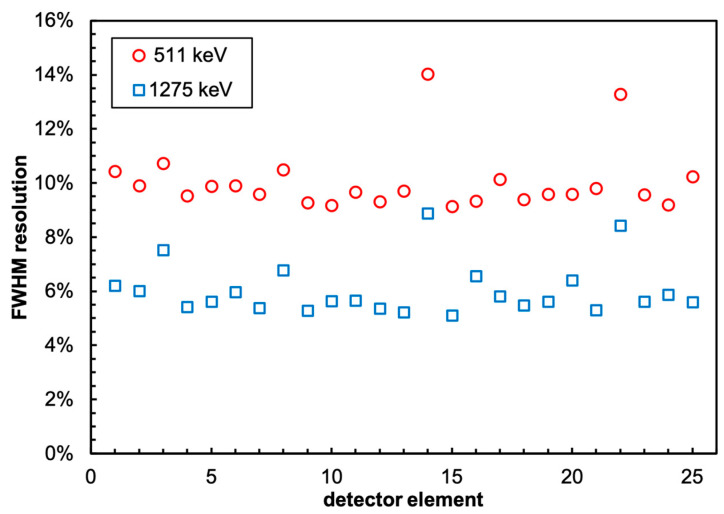
The energy resolution measured for the 25 detectors at 511 and 1275 keV.

The energy resolution of all 5 × 5 detectors of the sub-array was tested by placing a ^22^Na gamma source on top of the central element at 30 mm distance, with and without the collimator, and acquiring data for 20 min in both configurations. Figure 8, where the 25 calibrated spectra in the configuration without the collimator are plotted, shows that all of the detector elements behave quite similarly. The differences in counting rates are ascribed to the different geometrical efficiency levels because of the different distances from the source. A similar plot for the configuration with the collimator is presented in Figure 9, where, as expected, the overall counting rates are visibly suppressed, whereas the central detector rate decreases by roughly 50%. Noticeably, the collimated spectrum of the central element exhibits a structure at very low energy that is not present in the free spectrum, and which is likely due to the X-ray lines of Pb. The measured FWHM energy resolution of the 25 elements of the array was calculated by means of Gaussian fits of the peaks at 511 and 1275 keV, and the values are plotted in Figure 10. The average resolution values were 9.7% and 5.8%, respectively, in line with the typical values reported in the literature, apart from the two channels that presumably suffered from bad optical coupling or a lower SiPM gain.

**Figure 11:**
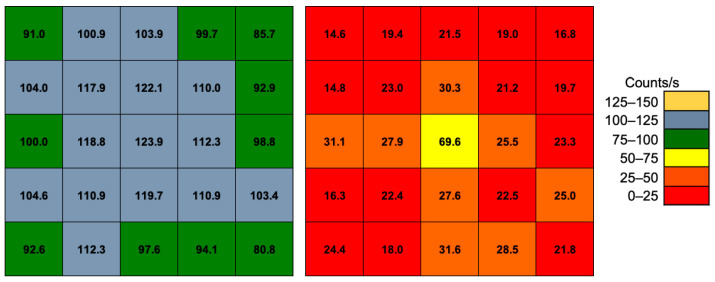
Overall count rates of the 5 × 5 array above a threshold of 30 keV. Left: without the collimator; right: with the collimator.

**Figure 12:**
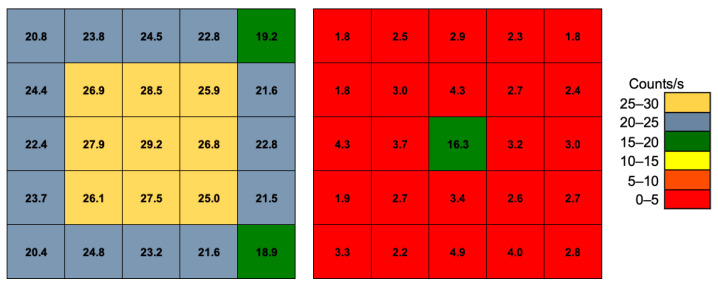
The count rates of the 5 × 5 array under the peak at 511 keV. Left: without the collimator; right: with the collimator.

**Figure 13:**
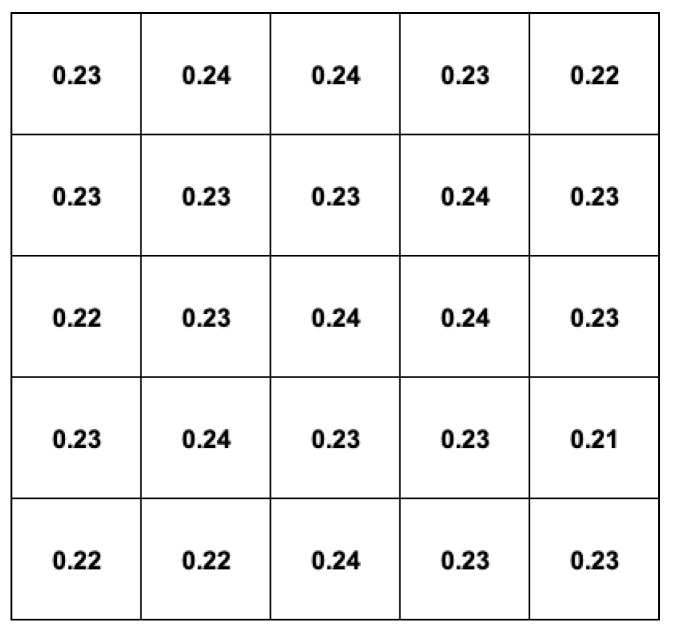
The peak-to-total efficiency ratio of each element, calculated as the ratio between the uncollimated count rates (left side) of Figure 11 and Figure 12.

By integrating the acquired spectra, the overall counting rate above a threshold of 30 keV was calculated for each detector channel, without and with the collimator. The results are reported in Figure 11. As expected, the effect of the collimator is quite evident as the ratio of the center-to-nearby counts goes from ≈1.1 without the collimator to ≈2.8 when the collimator is inserted. If the integration is limited to the region under the peak at 511 keV, the situation improves a lot, whereby the ratio of the center-to-nearby counts goes from ≈1.1 to ≈5.1, because mainly direct 511 keV gamma rays are taken into account, while Compton-scattered rays reaching the central cell are suppressed by the energy selection window (Figure 12). The peak-to-total efficiency ratio of each element, calculated as the ratio between the uncollimated count rates (left side) of Figure 11 and Figure 12, is reported in Figure 13.

**Figure 14:**
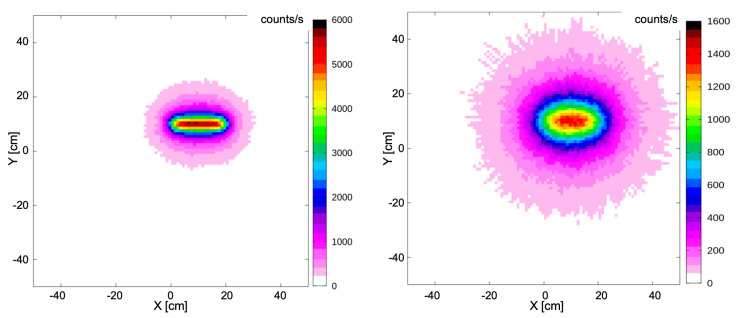
Hot-spot images of a 1 MBq ^137^Cs gamma source, uniformly distributed over a sheet measuring 2.5 × 20 cm^2^ placed at 1 cm (left) and a 5 cm (right) distances, obtained by simulating a table with 100 × 100 CsI(Tl) scintillators measuring 1 cm^3^.

In light of these promising results, the FLUKA code was used to perform two simulations in order to assess the possible performances of a larger table array [15]. A similar setup was simulated but made of 100 × 100 CsI(Tl) scintillators, in combination with a 1 MBq ^137^Cs gamma source uniformly distributed over a metal sheet measuring 2.5 × 20 cm^2^ placed at 1 cm distance from the table. The second simulation was done with the same setup and the source at 5 cm distance. The expected spatial distributions of the count rates for the two cases are shown in Figure 14.

## 4. Discussion

The results shown in the previous section can be briefly summarized with the following points:

- A modular flat array of CsI(Tl) scintillators with a SiPM readout can be reasonably built;
- It can be exploited using a quick and simple counting mode in order to determine the possible presence of hot-spots among the objects placed on top of it;
- It can provide a rough but effective proximity image, which is helpful in locating and identifying active objects;
- It can also provide space-resolved medium-resolution spectroscopic details, which are helpful in grossly identifying the present isotopes and possibly defining regions of interest (ROI) in the energy spectra to produce isotope-related images;
- In cases of low activity, several spectra can be summed up, thereby increasing the statistics at the price of lower (or null)-position information.

These features could pave the way for the construction of tables in several possible geometrical arrangements, according to different application needs. Indeed, the use of a suitable multichannel table detector, built according to our scheme, can facilitate this task. The presumably radioactive material can be placed on top of the table, providing a quick response in terms of the global counting rate and its spatial distribution. Should a finer analysis be needed, the table can provide the gamma spectrum measured by each element of the array.

An interesting application could be in the field of nuclear inspections [5,6], where samples and other materials are handled and exchanged between operators and inspectors. One could think of equipping the exchange desk with such an array capable of generating a prompt alarm in case of contaminated objects. Additional imaging and a spectroscopic analysis could then be automatically performed using a suitable software to identify the hot-spot before deciding whether an accurate check with a more sophisticated detector (e.g., a germanium detector) is needed.

A similar arrangement could be useful in the context of radioactive waste sorting and repackaging, which involves opening old drums and sorting their contents in terms of activity in order to reduce the radwaste volume. In this application, the waste would be placed on top of the array table that assesses the activity of the single objects and decides whether an object can be released or if it should be still classed as radioactive material and packed accordingly.

**Figure 15:**
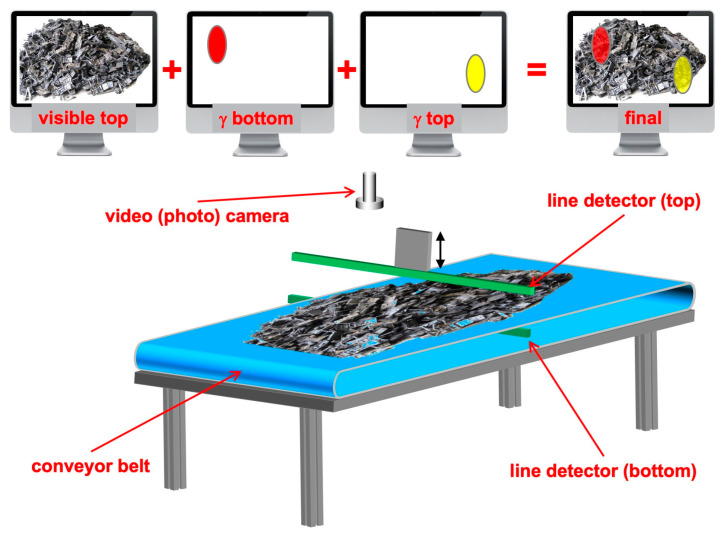
Sketch of a possible radwaste scanner based on a conveyor belt, a video camera and two linear arrays of detectors. The bottom array would be embedded in the conveyor, while the top one would be installed on an up–down actuator in order to be moved closer to the materials to be inspected.

Usage with objects on top of the table was assumed, and this is why distances of 1–2 cm were mainly considered, but one could use larger distances by possibly employing collimators for better-quality imaging. Several geometrical arrangements can be devised, being either rectangular and/or with more sparsely distributed scintillators, with a tradeoff between the space resolution and cost. One possible arrangement in particular is worth mentioning, i.e., a linear array in a fashion similar to the airport baggage scanners. This would represent an interesting tradeoff between the cost and scanning time, without sacrificing the space resolution. In Figure 15, a possible configuration is sketched of a radwaste scanner based on a conveyor belt, a video camera and two linear arrays of detectors. The bottom array would be embedded in the conveyor and the top one would be installed on an up–down actuator in order to be moved closer to the materials to be inspected. As an alternative, the conveyor belt could be replaced by a fixed surface and the two linear arrays could be moved to scan the observation field. The scan would be done line-by-line, in steps of 1 cm, and the data could be acquired during predefined time intervals or with variables as a function of the detected counting rates. Should a threshold be overcome, indicating the presence of a hot-spot, the operator could decide to perform a finer spectroscopic analysis.

Due to the CsI(Tl) scintillation light decay time of ≈3 µs, the maximum counting rate of each detection cell is of the order of 30–50 kHz, and this could be a limitation in cases where the objects have very high activity. Therefore, the future plans are to test a new configuration by replacing the CsI(Tl) array with one equipped with the faster BGO scintillator [16], whose emission wavelength, centered at 480 nm, is better matched to the spectral response of SiPM (PDE ≈ 25%). With such a scintillator, one could expect a similar energy resolution but a ten-fold faster 300 ns decay time that should permit the handling of much higher counting rates.

## 5. Conclusions

A small prototype of a gamma radiation detection system was described, based on CsI(Tl) crystals and SiPMs, which could be incorporated as part of a work desk to perform continuous monitoring of materials and to locate hot-spots. In addition, the system could perform a medium-resolution spectroscopic analysis of the materials under examination. The system is modular and can be replicated in many different geometrical arrangements according to the specific application needs and requirements. The individual elements of the array were characterized, and a possible configuration as a radioactive waste scanner was proposed. An alternative configuration capable of handling very high counting rates was also proposed based on the faster BGO scintillator, which will be tested in the near future.

## Figures and Tables

**Table 1:** The used laboratory sources, their activities and their full-energy peak gamma energies.

Source	Energy 1 [keV]	Energy 2 [keV]	Activity [kBq]
^241^Am	59	-	34.3
^22^Na	511	1275	66.1
^137^Cs	662	-	25.3
^60^Co	1173	1332	117

## Data Availability

The data presented in this study are available on request from the corresponding author.
